# Reversal of atherosclerosis by restoration of vascular copper homeostasis

**DOI:** 10.3389/ebm.2024.10185

**Published:** 2024-06-24

**Authors:** Xiao Zuo, Xueqin Ding, Yaya Zhang, Y. James Kang

**Affiliations:** ^1^ Tasly Stem Cell Biology Laboratory, Tasly Biopharmaceutical Co., Tianjin, China; ^2^ Regenerative Medicine Research Center, West China Hospital, Sichuan University, Chengdu, Sichuan, China

**Keywords:** atherosclerosis, copper, macrophages, inflammation, reversal therapy

## Abstract

Atherosclerosis has traditionally been considered as a disorder characterized by the accumulation of cholesterol and thrombotic materials within the arterial wall. However, it is now understood to be a complex inflammatory disease involving multiple factors. Central to the pathogenesis of atherosclerosis are the interactions among monocytes, macrophages, and neutrophils, which play pivotal roles in the initiation, progression, and destabilization of atherosclerotic lesions. Recent advances in our understanding of atherosclerosis pathogenesis, coupled with results obtained from experimental interventions, lead us to propose the hypothesis that atherosclerosis may be reversible. This paper outlines the evolution of this hypothesis and presents corroborating evidence that supports the potential for atherosclerosis regression through the restoration of vascular copper homeostasis. We posit that these insights may pave the way for innovative therapeutic approaches aimed at the reversal of atherosclerosis.

## Impact statement

Recently advanced understanding of pathogenesis of atherosclerosis transformed the disease treatment approach from slowing its progression to promoting the regression of atherosclerosis. Copper plays a critical role in the regulation of structural integrity and lipid metabolism of vascular tissue. However, copper is deficient in the atherosclerotic vasculature, and contrarily elevated in the blood of atherosclerotic patients. Experimental restoration of copper homeostasis between the vessel wall and the circulation reverses the established atherosclerosis in animal models. It is predictable that the time is coming for therapeutic reversal of atherosclerosis.

## Introduction

Atherosclerotic disease is a chronic inflammatory condition that affects the wall of arteries, resulting in a buildup of plaque and subsequent narrowing or blockage of blood vessels [1, 2]. Atherosclerosis is responsible for a range of cardiovascular and cerebrovascular diseases, such as heart attack, heart failure, and stroke. Atherosclerosis has been a major contributor to the global burden of cardiovascular disease, which remains one of the leading causes of mortality worldwide [3].

**FIGURE 1:**
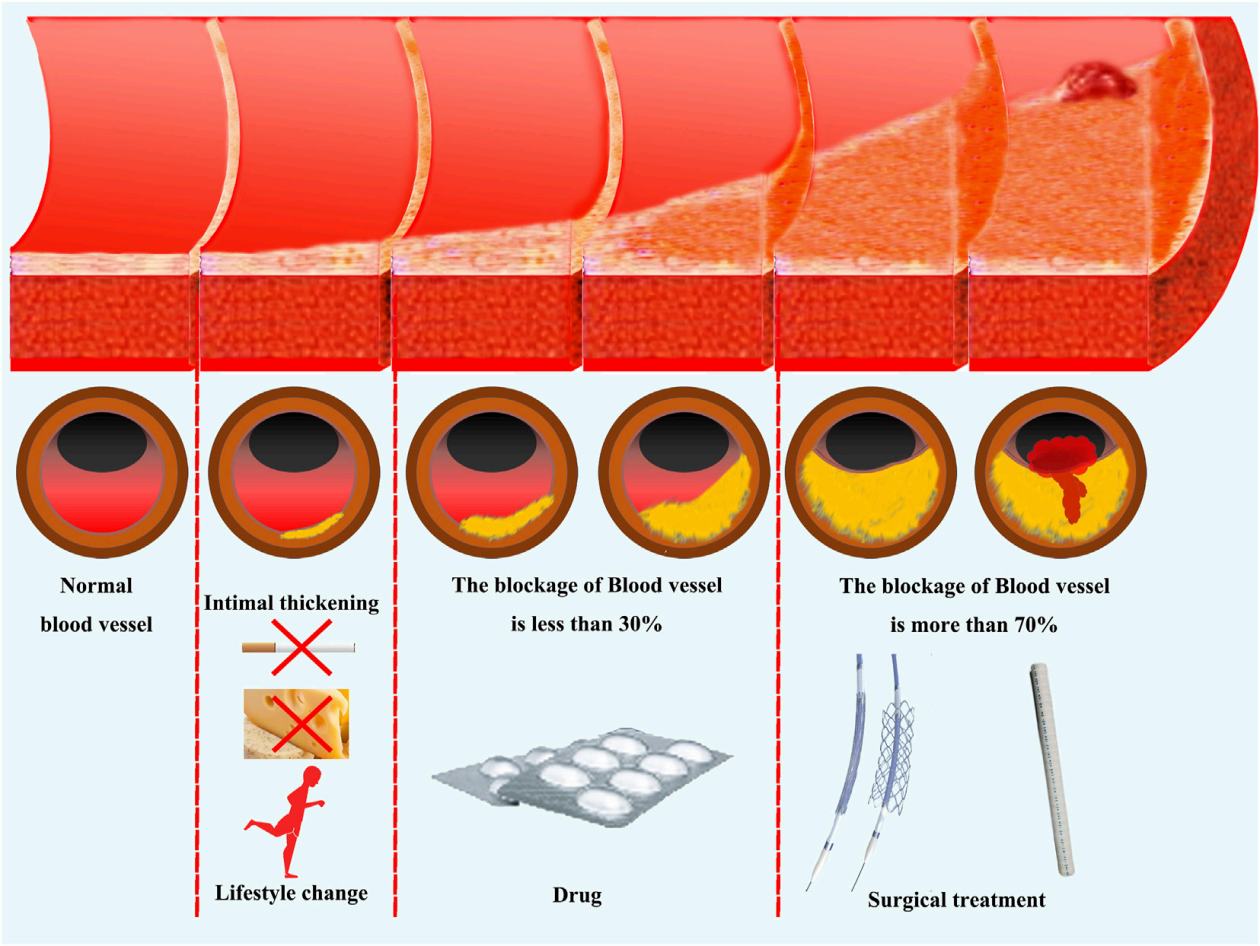
Current clinical approaches for the treatment of atherosclerosis. Antagonistic therapy against pathogenic factors is the mainstay of currently clinical treatments for atherosclerosis. In the early stages of vascular lesions, disease risk factors can be reduced by changing one’s lifestyle. When the thickening of the vascular endothelium and the degree of vascular obstruction is less than 30%, the disease progression can be controlled through drug therapy. When vascular obstruction exceeds 70% or patients exhibit significant clinical symptoms, revascularization treatment can be performed through surgery.

Currently, atherosclerosis is widely considered as a progressive and irreversible disease [4, 5]. As summarized in Figure 1, clinical therapies primarily involve antagonistic therapy approaches that focus on inhibition of factors contributing to the occurrence and progression of the disease, with the aim of delaying the progression of the disease and reducing the risk of cardiovascular events due to plaque rupture [6].

Primary therapeutic interventions for atherosclerosis includes lifestyle modifications, such as regular exercise, a healthy diet, smoking cessation, and management of hypertension, diabetes, and high cholesterol levels [7, 8]. Such alterations in lifestyle can exert preventative effects at the nascent stages of the disease, potentially restoring some patients to a state of health [9–11]. However, these lifestyle modifications fail to reverse the condition once the disease has progressed to the phase characterized by intimal thickening within the blood vessels [12]. At this juncture, pharmacological interventions become the cornerstone of atherosclerosis management.

Statins are commonly prescribed for effective reduction of blood cholesterol levels and lowering the risk of plaque formation [13, 14]. Furthermore, antiplatelet agents, such as aspirin, are employed to inhibit blood clot formation within arteries [15, 16]. Moreover, medications including angiotensin-converting enzyme (ACE) inhibitors and angiotensin receptor blockers (ARBs) are prescribed to address hypertension, which often concomitantly exist with atherosclerosis, thus contributing to a comprehensive management strategy for the condition [17].

Inflammation is known to play a decisive role in the progression of atherosclerosis. Therefore, inhibition of inflammation is currently considered as the most important treatment option [18–21]. While early administration of broad-spectrum anti-inflammatory and anticoagulant drugs, such as aspirin and colchicum, can reduce the risk of cardiovascular events, these approaches, however, do not have a significant effect on regression of the disease condition [22]. In addition, there are considerable risks associated with broad-spectrum anti-inflammatory drugs, such as tumorigenesis [23].

It appears that, although current drug therapy can delay the progression of atherosclerosis to some extent, the diseased vessels remain prone to blockage or atherosclerotic plaque rupture. Surgical treatment is typically reserved for patients with advanced stage of atherosclerosis, particularly when the vascular obstruction surpasses 70%, or when patients exhibit significant clinical symptoms including pain or functional impairments in the distal extremities, indicative of inadequate blood flow [24]. The most common surgical procedures for treating advanced atherosclerosis include angioplasty, stent placement, and coronary artery bypass grafting (CABG).

Angioplasty entails the introduction of a balloon catheter into a constricted artery, followed by balloon inflation to expand the arterial passage [25]. In stent placement is to use a small metal mesh tube implanted within the artery to hold it open [26]. CABG involves the transplantation of a healthy blood vessel to circumvent the obstructed segment of the artery [27]. These interventions aim to restore blood flow by physically clearing the blockages. However, despite the effectiveness of these efforts, the issue of progressive stenosis and re-occlusion of blood vessels remains a significant challenge, putting patients at risk of secondary surgery or acute vascular occlusion [28, 29].

Therefore, combating atherosclerosis is a challenging task. There is an urgent need for a new approach focusing not only on delaying the progression of the disease, but also on fundamentally restoring the structure and function of the diseased blood vessels. To achieve this goal, it is crucial to establish new treatment strategies from the perspective of promoting vascular repair and regeneration. These strategies should be based on more advanced understanding of the pathological mechanisms of atherosclerosis.

In this review, we summarize the advanced knowledge of the pathogenesis of atherosclerosis, drawing attention to the role of copper metabolism in maintaining vascular homeostasis. We analyzed the potential of restoring copper homeostasis in atherosclerotic plaque tissue to reverse the progression of atherosclerosis. We also provided an overview of the current research in this area and discussed the challenges that need to be addressed for future development of clinically feasible treatments for the reversal of atherosclerosis.

## The pathogenesis of atherosclerosis

**FIGURE 2:**
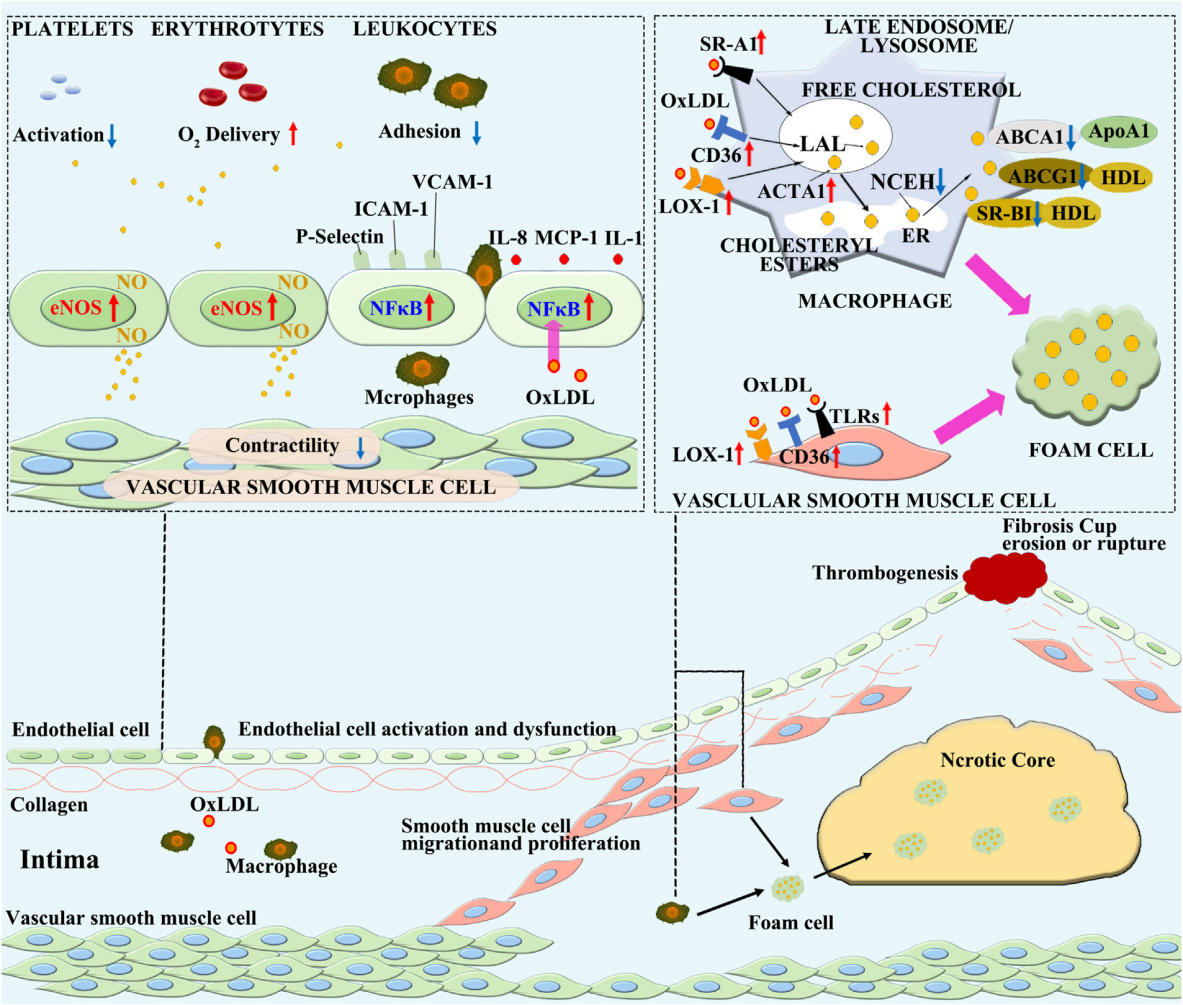
The pathogenesis of atherosclerosis. Various pathological factors in the blood cause dysfunction of ECs, leading to lipid deposition in the vascular intima. The oxidation of lipids to oxidized lipoproteins constitutes an initial inflammatory microenvironment, which further stimulates the activation and inflammatory transformation of ECs. Monocytes in the blood are attracted into the vascular intima by inflammation, where they differentiate into macrophages. In the inflammatory microenvironment, the steady state lipid metabolism in macrophages is disrupted, leading to the engulfment of oxLDL without restriction. On the other hand, the ability of macrophages to excrete lipids is decreased, resulting in lipid accumulation within macrophages and the formation of foam cells. Inflammatory macrophages exacerbate further development of inflammation, and with the accumulation of a large number of foam cells, cell necrosis occurs, forming a necrotic core. Smooth muscle cells are activated by inflammatory stimuli and form a fibrous cap on the surface of the plaque by migration, proliferation, and secretion of collagen, leading to the formation of a stable plaque. As inflammation continues to intensify, the necrotic core continues to enlarge, smooth muscle cells undergo massive necrosis, and the collagen fibrous cap becomes thinner and vulnerable. Ultimately, the plaque ruptures, leading to thrombosis.

Atherosclerosis is a progressively pathological process characterized by chronic inflammation as its primary pathogenic mechanism [21, 30]. The stages of its initiation and development are outlined below and diagrammed in Figure 2.

### Endothelial cell dysfunction and lipid particles accumulation

The vascular endothelium plays a critical role in regulating homeostatic network of the cardiovascular system [31]. Endothelial cell dysfunction (ECD), which marks the initial alteration in the trajectory of atherosclerotic lesion progression, is typified by the activation of endothelial cells (ECs) and their shift from an anti-inflammatory to a pro-inflammatory state [32, 33]. Recent studies suggest that the hemodynamic change is the primary cause of ECD [34].

Under undisturbed laminar flow, ECs exhibit an up-regulation of Kruppel-like factor 2 (KLF2), a transcription factor integral to maintain vascular equilibrium. This upsurge in KLF2 expression precipitates an increase in endothelial nitric oxide synthase (eNOS) gene expression, catalyzing the synthesis of nitric oxide (NO). NO, a small lipid soluble molecule, traverses cell membranes to influence a wide array of cell functions within the bloodstream and the vascular endothelium [35–37]. NO’s effects include the inhibition of platelet activation, adhesion, and aggregation [38], a reduction in leukocyte adhesion to the endothelium [39], a facilitation of vasorelaxation through dephosphorylation of myosin light chain in vascular smooth muscle cells [40], and an enhancement of the oxygen delivery capacity of red blood cells [41].

Steady laminar flow promotes the alignment of ECs [34] and inhibits signal transduction by pro-inflammatory stimuli such as TNF and interleukin-1 (IL-1) [42]. This hemodynamic condition diminishes IL-6 induced progression of cell cycle in ECs [43], and protects against ECs apoptosis, ensuring the endothelial integrity [44].

Blood flow irregularities trigger ECs activation, leading to a consequential decrease in eNOS gene expression and the stimulation of nuclear factor kappa B (NF-κB) signaling pathway [45, 46]. NF-κB plays a pivotal role in the initiation of pro-inflammatory responses within the endothelium. These responses include up-regulation of ECs surface adhesion molecules including vascular cell adhesion molecule-1 (VCAM-1); the release of chemokines such as monocyte chemoattractant protein-1 (MCP-1) and Fractalkine; and the production of pro-thrombotic mediators including tissue factor (TF), von Willebrand factor (vWF), and plasminogen activator inhibitor-1 (PAI-1), in both their soluble and membrane-bound forms [47–49].

Activated ECs cause an increased production of reactive oxygen species (ROS) [50]. The resulting oxidative stress, in addition to prolonged inflammation, disrupts adherent junctions, such as VE-cadherin and gap junctions, primarily due to the reduction in NO levels [51]. This disruption leads to an increased accumulation of sub-endothelial atherogenic apolipoprotein B (ApoB)-containing lipoproteins, including LDL, VLDL and chylomicrons [52, 53]. Importantly, the increased ROS facilitates oxidative modification of ApoB-containing lipoproteins [54], which act as dual-function agents in the immune response: serving as antigens that initiate the adaptive immune response and as adjuvant molecules that stimulate the innate immune system [55–57].

Lifestyle modifications and pharmacological interventions can improve the blood microenvironment by lowering lipid levels, restoring EC’s function. If ECs function is not restored, the lipid deposition within the vessel wall will persist. The retention of oxidized low-density lipoprotein (oxLDL) in the intima of blood vessels acts as a constant source of inflammatory stimulus, continuously activating ECs and triggering inflammatory responses [58, 59]. Therefore, it is crucial to remove oxLDL to suppress the pathogenesis of atherosclerosis.

### Immune cell recruitment and foam cell formation

Macrophages are the major immune cells that respond to inflammation triggered by oxLDL, playing a pivotal role in the formation and progression of atherosclerotic lesions [55, 57]. Macrophages mainly originate from myeloid progenitor cells in the bone marrow. Myeloid progenitor cells develop into circulating monocytes, which can infiltrate into atherosclerotic lesions from the bloodstream or from the spleen that acts as a reservoir for monocytes in mice [60]. The recruitment of monocytes to atherosclerotic lesions is mediated by the activation of ECs.

Activated ECs initiate monocyte recruitment by enabling their initial rolling on the endothelium through P-selectin engagement, followed by the strengthening of monocyte adhesion via interactions with immunoglobulin-G family proteins, VCAM-1 and ICAM-1. Subsequent monocyte infiltration into the subendothelial layer is driven by chemokines such as MCP-1 and IL-8 [35, 61, 62]. Once penetrating the intima of blood vessels, monocytes encounter a pro-inflammatory milieu constructed by activated ECs and oxidized lipid particles. This environment fosters the transformation of monocytes into pro-inflammatory macrophages, actively up-taking lipids [63, 64].

Macrophages play a crucial role in regulating plasma lipoprotein content and metabolism [65]. Under normal conditions, macrophages recognize native LDL via the LDL receptor (LDLR). The LDL is then endocytosed and transported to lysosomes, where the cholesteryl ester (CE) is hydrolyzed to free cholesterol (FC) by acid lipase [66, 67]. The FC is then transferred to the endoplasmic reticulum (ER) to be esterified by acyl CoA:cholesterol acyltransferase 1 (ACAT1). The CE produced by ACAT1 is stored in cytoplasm as lipid droplets, undergoing a continual cycle of hydrolysis to FC by neutral cholesterol esterases and re-esterification by ACAT1 [68, 69].

Neutral cholesteryl ester hydrolase 1 (NCEH1) processes CE, releasing FC that is transported out of the cell through ATP-binding cassette (ABC) transporters, including ABCA1 and ABCG1 and scavenger receptor class B1 (SR-BI) [70]. Apolipoprotein A-1 (ApoA-1) acts as a receptor for cholesterol transported by ABCA1, while high-density lipoprotein (HDL) accepts cholesterol transferred by ABCG1 and SR-BI. This machinery is tightly regulated under normal conditions to maintain cholesterol homeostasis. An increase in FC in an ER regulatory pool triggers a signaling cascade that down-regulates the LDL receptor, preventing foam cell formation in hypercholesterolemia. Thus, these proteins ensure an effective control of LDL and cholesterol content in peripheral blood under normal conditions [71–73].

In atherosclerosis, the process of macrophage-dependent cholesterol handling is disrupted. In addition to an increase in the production of oxLDL, macrophages are stimulated by multiple inflammatory factors and express various scavenger receptors, including SR-A1, CD36, and lectin-like oxLDL receptor-1 (LOX-1), which all have an affinity for oxLDL [74, 75], leading to an excessive uptake of oxLDL transforming macrophages to foam cells [76]. Simultaneously, the activity of ACAT is elevated, resulting in an overproduction of CE that accumulate in the endoplasmic reticulum. Furthermore, the expression of NCEH, ABCA1, and ABCG1 is decreased in atherosclerosis, further exacerbating intracellular cholesterol accumulation and foam cells formation [77, 78].

In addition to monocyte-derived macrophages, vascular smooth muscle cells (VSMCs) also significantly contribute to the foam cell population [79]. Clinical studies found that over 50% of foam cells may be derived from VSMCs in human atherosclerotic lesions [80]. Intracellular cholesterol accumulation inhibits VSMC gene expression (including α-smooth muscle actin (α-SMA), smooth muscle myosin heavy chain (SMMHC), and smooth muscle 22α (SM22α) and induces the expression of pro-inflammatory and macrophage markers [81]. VSMCs uptake oxidized LDL mainly through LDLR family and SR family [75, 82, 83]. Compared to macrophages, VSMCs are inefficient at lysosomal processing and cholesterol trafficking, with a much low expression of ABCA1 [84], contributing to an impaired cholesterol efflux [85]. In addition, ECs and dendritic cells have also been reported to participate in the formation of foam cells [86].

In short, lipids infiltrated in the vascular intima are engulfed by macrophages, smooth muscle cells, and other cells, and eliminated through lipid metabolism pathways. However, in the pathogenesis of atherosclerosis, the balance between lipid engulfment and elimination by the effector cells is disrupted. The excessive engulfment of oxLDL leads to lipid accumulation in these cells transforming these cells to foam cells. This progression pushes the atherosclerosis lesion towards a more severe and uncontrollable direction.

### Necrotic core formation and plaque rupture

During the process of intravascular adipose streaks formation or pathological intimal thickening, inflammatory ECs and macrophages attract more monocytes to infiltrate the intima by secreting chemokines such as CCR2, CCR5, CXCR1, and CXCR2 [87, 88]. In the early stage of vascular intimal lesions, monocyte infiltration and aggregation are the main driving factors, while in later stages, macrophage proliferation becomes more important [89]. Inhibition of macrophage proliferation has been shown to reduce the size of plaque [90].

Within the inflammatory milieu, macrophages and other immune cells further release inflammatory factors, such as transforming growth factor-β (TGF-β), platelet-derived growth factor (PDGF) isoforms, matrix metalloproteinases (MMPs), fibroblast growth factor (FGF), and heparin-binding epidermal growth factor (HB-EGF), activating VSMCs located in the arterial wall media [91, 92]. Activated VSMCs gain the ability to proliferate, migrate, and secrete various extracellular matrix (ECM) proteins, and are attracted to the lesion to form a fibrous cap that stabilize lipid plaques [93].

The formation of the fibrous cap makes the atherosclerotic lesion less reversible. The persistence of inflammation leads to the accumulation of apoptotic cells that cannot be cleared by macrophages in a timely manner, causing secondary necrosis. This in turn further promotes inflammation, oxidative stress, and death of adjacent cells, forming a necrotic core [94–96].

The growth of the necrotic core causes a thinning of the fibrous cap. The accumulation of inflammatory cytokines and oxidative products in the vascular lesion provoke uncontrolled accumulation of adjacent VSMCs and, consequently, decreased synthesis of ECMs [96–98]. The components of the ECMs are degraded by macrophage-derived MMPs [99, 100], elastase, and tissue protease [101]. Under this condition, the production of TGF-β is reduced, leading to a decrease in collagen production in healthy VSMCs [102, 103]. In combination, these factors accelerate the thinning of the fibrous cap.

Core necrosis and fibrous cap thinning transform stable fibrous plaques to vulnerable plaques. Plaque rupture abruptly exposes the plaque interior to circulating pro-coagulant factors and platelets, leading to thrombosis [104, 105]. Atherosclerosis-associated clinical events are mainly attributed to thrombus detachment, causing acute vascular occlusion in major organs, leading to myocardial infarction, pulmonary embolism, and stroke.

## Copper regulation of vascular metabolism and function

Copper (Cu) is an essential mineral nutrient that participates in cellular metabolism and function as a component of a number of cuproenzymes, an integrated structural element, and a regulatory agent [106–108]. However, Cu also catalyzes the production of highly reactive oxygen species (ROS), which have the potential to cause oxidative damage to lipids, proteins, DNA and other molecules [109–111]. Therefore, either Cu deficiency or excess can lead to diseases or affect the progression of diseases including atherosclerosis. Understanding the complexity of the role of Cu in vascular homeostasis is helpful in designing targeted therapies for reversal of atherosclerosis.

### Cu promotion of angiogenesis

The involvement of Cu in angiogenesis has been known for more than 40 years [112, 113]. In 1980s, studies using rabbits demonstrated that Cu and Cu-binding proteins significantly induced angiogenesis in the cornea [113]. It was further found that CuSO_4_ alone stimulated the expression of VEGF in human keratinocytes in a dose-dependent manner [114]. Following these observations, a series of studies revealed that Cu is instrumental in regulating various EC functions, including proliferation, migration, and tube formation [115–117]. Cu-binding proteins play a critical role in VSMCs migration [118–120] and blood vessel maturation [115, 121]. Mechanistic understanding of the role of Cu in angiogenesis during last two decades revealed that Cu promotion of angiogenesis acts through its regulation of hypoxia-inducible factor-1 (HIF-1) in multiple cell types [122, 123].

**FIGURE 3:**
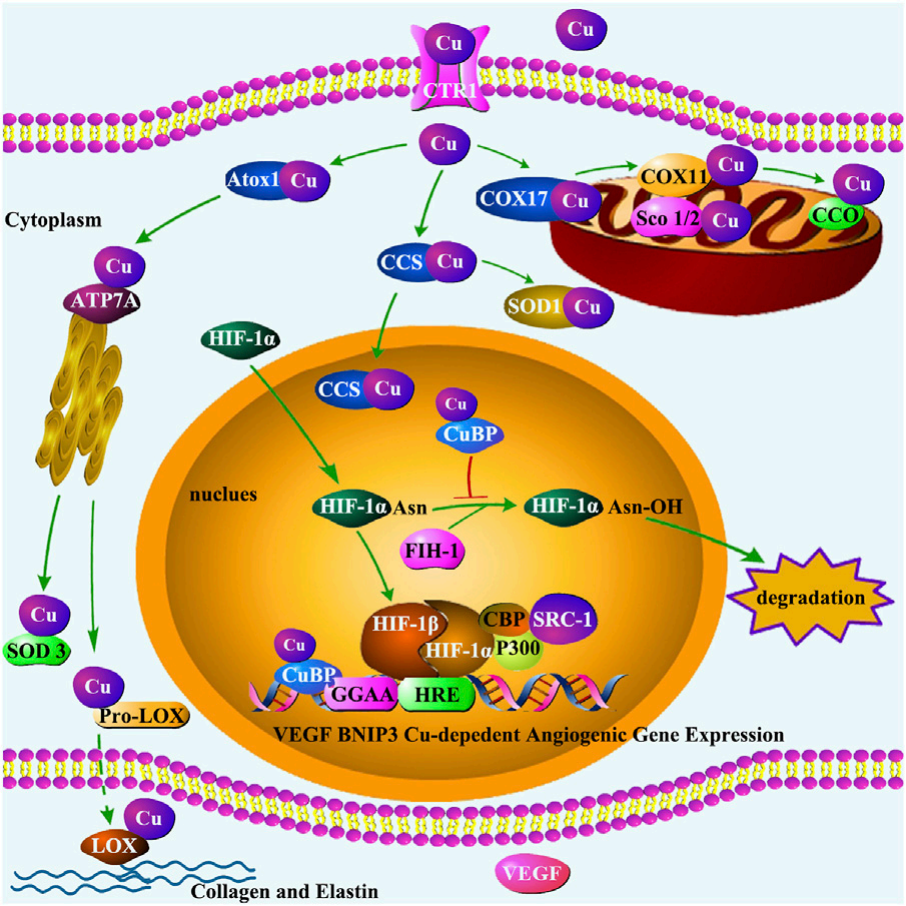
Cu trafficking in the cell and its regulation of HIF-1 transcriptional activity. The transportation of Cu by CTR1 enables Cu chaperones to acquire Cu, transferring Cu to Cu-containing enzymes and proteins. Three primary pathways have been identified for Cu chaperones: (1) delivering Cu to CCO in mitochondria by Cox17 and Sco1/Sco2 proteins; (2) delivering Cu to SOD1 in the cytosol and mitochondrial intermembrane by CCS; and (3) delivering Cu to secretory Cu enzymes such as extracellular SOD3 and LOX by Atox1 via Cu transporter ATP7A located in the trans-Golgi network. SOD3 protects cells by scavenging ROS. LOX regulates the formation of ECM, playing an important role in maintaining vascular homeostasis. In addition, under hypoxia condition, CCS transports Cu to the nucleus, where HIF-1α dimerizes with HIF-1β. The HIF-1 heterodimer recruits cofactors such as p300/CBP and SRC-1 to form transcriptional complex, a process may be inhibited by FIH-1. Cu-binding proteins (CuBP) act as an inhibitor for FIH-1activation to ensure the formation of HIF-1 transcriptional complex. The interaction of HIF-1 with HRE requires Cu to initiate the Cu-dependent expression of genes such as VEGF and BNIP3. VEGF plays an important role in promoting endothelial cell proliferation. The core base “GGAA” (the core motif of the ETS family) is a crucial motif in the binding site of Cu-dependent genes.

The processes of Cu trafficking between intracellular organelles are depicted in Figure 3, including the indications of Cu regulation of HIF-1 transcriptional activity for angiogenetic gene expression. The initial evidence of Cu interaction with HIF-1 was reported by Jiang et al., who explored how dietary Cu supplementation mitigates pressure overload-induced cardiac hypertrophy in mice [124]. Sustained cardiac pressure overload leads to reduced myocardial Cu and VEGF levels, and diminished angiogenesis. Cu replenishment increases VEGF and promotes angiogenesis in the hypertrophic hearts, leading to regression of cardiac hypertrophy. Further studies found that in cultured human cardiomyocytes, Cu chelation blocks insulin-like growth factor (IGF)-1- or Cu-stimulated VEGF expression, which is relieved by addition of excess Cu. Both IGF-1 and Cu activate HIF-1α. Consequently, HIF-1α gene silencing blocks IGF-1- or Cu-stimulated VEGF expression. In addition, HIF-1α coimmunoprecipitates with a Cu chaperone for superoxide dismutase-1 (CCS-1), and gene silencing of CCS-1 prevents IGF-1- or Cu-induced HIF-1α activation and VEGF expression [124]. Thus, Cu promotion of angiogenesis is mediated by HIF-1α activation of angiogenic gene expression with the aid of CCS-1.

Cu regulates HIF-1 transactivation of angiogenic gene expression in multiple mechanisms of action. Under hypoxic conditions, Cu enters the nucleus in both CCS-1-dependent and -independent processes [125]. In the cytoplasm, Cu stabilizes HIF-1α, the rate-limiting component of HIF-1, leading to its accumulation and promoting its entrance to the nucleus [126–128]. In the nucleus, Cu inhibits the activity of asparaginyl hydroxylase factor inhibiting HIF-1 (FIH-1) and ensures the formation of HIF-1 transcriptional complex [129]. Importantly, Cu selectively regulates the binding of HIF-1 to the HRE elements of target angiogenic genes by affecting the interaction between the transcription factor and the promoter region of the angiogenic genes [130, 131].

Cu selectively regulates the process of HIF-1 transactivation of angiogenic gene expression. It is important to note that not all of the HIF-1 regulated genes require Cu for expression [130, 132]. This was first demonstrated by an *in vitro* study in which the treatment of HUVECs with a Cu chelator, tetraethylenepentamine (TEPA), suppressed the expression of a group of HIF-1 target genes such as BNIP3 and VEGF, but did not affect other HIF-1 target genes such as IGF-2 [132]. This Cu selective regulation of the expression of HIF-1-controlled genes was further defined in studies of monkey model of HIF-1 regulation of angiogenesis in ischemic myocardium [133]. During the acute phase of ischemic injury, angiogenesis was activated in the injured heart along with an increase in angiogenic factors. In the chronic phase of myocardial ischemia, sustained accumulation of HIF-1α was observed. However, the accumulation of HIF-1α was not accompanied by the expression of HIF-1-controlled angiogenic factors, including VEGF, tyrosine-protein kinase receptor Tie-2, angiopoietin-1 (Ang-1), and FGF-1 in the ischemic myocardium [133]. On the other hand, an up-regulation of HIF-1-controlled non-angiogenic gene expression such as IGF-2 was associated with HIF-1α accumulation [133].

This paradoxical phenomenon, HIF-1α accumulation being accompanied by suppression of HIF-1 target angiogenic gene expression, is now recognized to be ascribed to the reduced Cu concentrations in the ischemic heart [133]. In response to ischemic insult, Cu content in the heart is significantly decreased, along with a significant increase in serum Cu concentrations [134–136]. A recent study using ChIP-sequencing and RNA-sequencing identified 218 Cu-dependent and 10 Cu-independent HIF-1 target genes across the genome under hypoxic conditions [131]. Cu efflux from the heart under hypoxic conditions leads to suppressed expression of Cu-dependent HIF-1 target genes, but does not change the expression of Cu-independent HIF-1 target genes.

The mechanism by which Cu selectively regulates the binding site of the HIF-1 target genes was recently revealed by a study in HUVECs [130]. In this study, Cu deprivation by TEPA completely suppressed the binding of HIF-1α to HRE site of BNIP3 along with a complete inhibition of BNIP3 mRNA expression, but the binding of HIF-1α to the HRE site of IGF-2 or the expression of IGF-2 mRNA was not affected under hypoxic conditions. Furthermore, *de novo* motif analysis of all 218 Cu-dependent and 10 Cu-independent HIF-1 target genes further revealed that the core bases “GGAA” and “TTCC,” previously identified as the core motifs for E26-transformation-specific (ETS) family [130] constitute the critical motifs for the binding sites of Cu-dependent genes, while there is no specific motif found in Cu-independent genes except the motif for HIF-1α [130]. The difference in the binding loci and pattern between all the Cu-dependent and Cu-independent HIF-1 target genes indicate that Cu, by selectively affecting the binding of HIF-1α to the critical motifs in the promoter and putative enhancer regions of HIF-1-regulated genes, selectively regulates the expression of HIF-1-controlled angiogenic genes.

### Cu regulation of endothelial cells (ECs)

**FIGURE 4:**
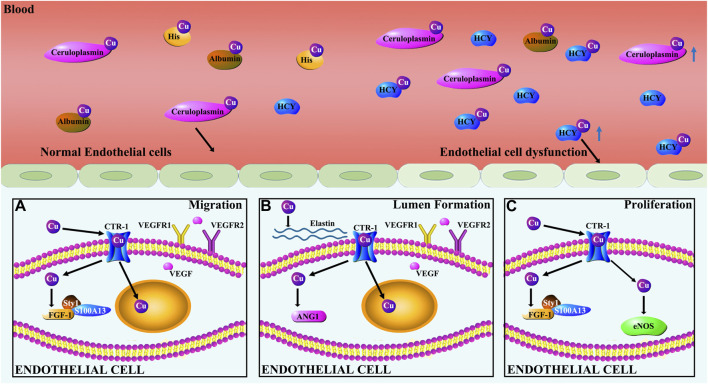
Cu regulation of endothelial cells. **(A)** Cu induces migration of ECs by regulating the expression of FGF-1 and VEGF. Specifically, Cu stimulates the release of FGF-1 from ECs by promoting FGF-1 binding to S100A13 and p40Syt1. Cu promotes HIF-1 transcriptional activity leading to increased VEGF gene expression. VEGF, in turn, regulates the dynamic transition of ECs by controlling the relative levels of VEGFR1 and VEGFR2. **(B)** Cu modulates EC-involved lumen formation by promoting the action of ANG-1 and elastin. ANG-1 is involved in vasculature stability, tightening cell contacts, inducing pericyte adhesion, and preventing alterations in vessel permeability. **(C)** Cu promotes proliferation of ECs by activating eNOS and FGF-1.

The regulatory action of Cu on ECs, as depicted in Figure 4, promotes angiogenesis. Cu at physiologically relevant levels stimulated vessel tube formation from HUVECs cultured in Matrigel [115], indicating the involvement of ECs proliferation, migration and integration in this process. ECs migration is a critical process in the vessel tube formation. Cu stimulation of ECs migration was confirmed using HUVECs for wound healing and transwell migration assays [115]. In the process of Cu stimulation of ECs migration, a Cu transporter-1 (CTR-1) is critically involved. Gene silencing by siRNA targeting CTR-1 in HUVECs significantly suppressed Cu entrance to the cells along with an inhibition of ECs migration [115].

Cu promotes ECs proliferation that is essential for the maturation of new blood vessels. Recent studies showed that Cu stimulates ECs proliferation in a concentration-dependent fashion within the effective concentration range [115–117]. NO generated by eNOS plays a critical role in regulating ECs proliferation, angiogenesis and vascular homeostasis [117]. Cu stimulation of ECs proliferation is eNOS-dependent [117]. Li et al found that Cu increased the expression of eNOS in HUVECs and that siRNA targeting eNOS blocked Cu stimulation of ECs proliferation [117].

Exposure of ECs to excessive Cu stimulates the expression of pro-inflammatory cytokines in the cells, leading to endothelial dysfunction [35, 137]. Excessive Cu accumulation in the blood was found in the pathogenesis of atherosclerosis. It is important to note that under the condition of atherosclerosis, Cu is deficient in the atherosclerotic lesion walls, but Cu concentrations are increased in the circulation [138–142]. The endothelial dysfunction is closely related to the disturbance in Cu homeostasis between the vascular wall and circulation, being the critical event in the initiation and progression of atherosclerosis [32, 33, 143].

Serum Cu elevation is closely associated with hyperhomocysteinemia [144]. Experimental and clinical studies over the last decade have shown that the elevation of blood homocysteine (Hcy) levels is linked to increased risk of atherosclerosis [145, 146]. Patients with homocystinuria were associated with high plasma Cu concentrations [144, 147, 148]. A correlation between plasma concentrations of total Cu and Hcy was identified [149, 150]. Addition of small amounts of Cu significantly enhanced the inhibitory effect of Hcy on ECs function, thus suppressing angiogenesis in isolated endothelial tissues in culture [151]. Cu and Hcy complexes have been identified *in vitro* and their exposure to cultured ECs elicited remarkable changes in relation to atherogenic activities [152–155].

### Cu regulation of lipid metabolism

There is increasing evidence that indicates a strong correlation between Cu homeostasis and lipid metabolism. Systemic Cu alterations appear to be inversely correlated to the level of lipids and lipid-transporting lipoproteins in the peripheral circulation [156, 157]. It was observed that Cu-deficient diet feeding induced Cu deficiency in organ systems in rats along with increased levels of circulating HDL and LDL, and an increase in total cholesterol, triglyceride and phospholipid levels [158, 159]. The total volume of HDL components in the blood was elevated in Cu deficient Sprague-Dawley rats, accompanied by a corresponding increase in the cholesterol and protein content of the HDL and LDL fractions [159, 160]. In Cu deficient rats, the triglyceride content of circulating LDL and VLDL was increased, as was the ApoE content of HDL [159, 160], however, the concentration of liver cholesterol was reduced [161]. Systemic Cu deficiency is associated with an increased production of HDL and an increased turnover of HDL cholesterol esters [159].

The polarity of macrophages affects the role that they play in the regulation of lipid metabolism [162]. Cu in its Cu^2+^ ionic form was found to play an important role in the regulation of macrophage polarization [163]. The concentrations of Cu^2+^ lower than 10 μM promoted the expression of M2 related genes in macrophages. However, higher concentrations of Cu^2+^ (100 μM) stimulated pro-inflammatory marker expression [163]. This bipolar regulation of macrophages by Cu ions is of a significant impact on the role of macrophages in the initiation and progression of atherosclerosis. In particular, the elevation of Cu concentrations in the blood in the progression phase of atherosclerosis would affect the severity of atherosclerotic lesions.

The oxidative modification of LDL is a key event in human atherosclerosis. Cu ions catalyze oxidative modification of LDL *in vitro* [164–166]. It was also reported that Cu participates in the oxidation of LDL *in vivo* [167]. It was found that high cholesterol feeding leads to Cu deficiency in the vessel tissue and Cu elevation in the plasma [138]. The increase in plasma Cu associated with high cholesterol feeding would promote oxidative modification of LDL, thus promoting atherosclerosis. HDL is more sensitive to oxidation by Cu than LDL [168, 169]. Dose-dependent oxidative damage to HDL and protective effect of vitamin E against oxidation of HDL was observed in the studies of Cu incubation with HDL [170].

Cuproptosis, a newly identified Cu-induced cell death, occurs via Cu binding to lipoylated enzymes in the tricarboxylic acid cycle, leading to subsequent protein aggregation, proteotoxic stress, and eventual cell death [171]. It is possible that high levels of plasma Cu under the condition of atherosclerosis cause cuproptosis of macrophages and other immune cells in the circulation, leading to unbalanced immunological responses in the circulation. However, cuproptosis may not take place in the endothelial cells because they cells are Cu deficient under the condition of atherosclerosis [138, 139]. This phenomenon, high circulation versus low tissue Cu levels, underscores the critical role of Cu homeostasis in health and diseases, as discussed in several recent reviews [172–174].

### Cu regulation of extracellular matrix

**FIGURE 5:**
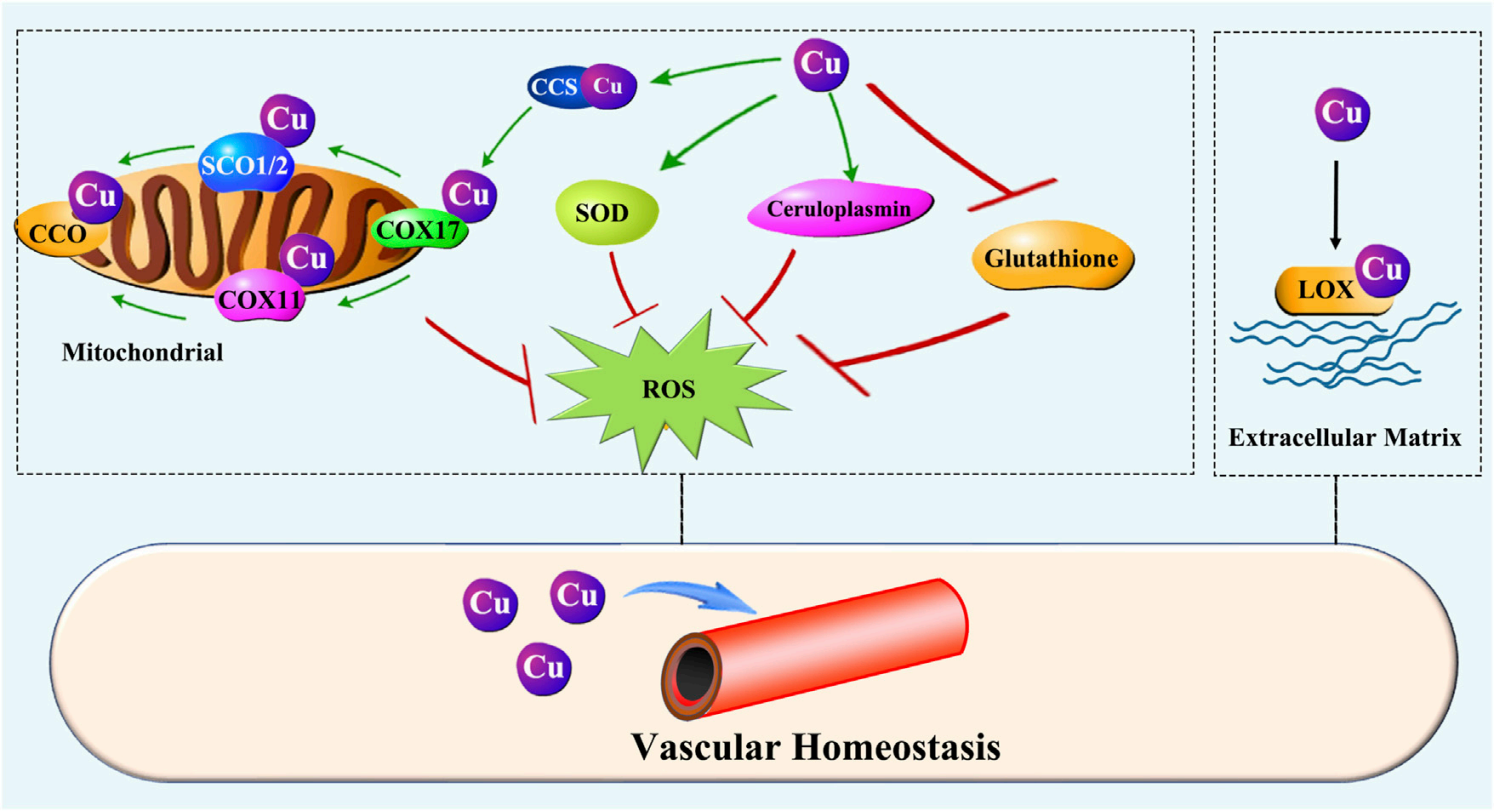
Cu regulation of vascular homeostasis. Cu prevents oxidative injury in the vascular system by ensuring the activity of key enzymes such as SOD, ceruloplasmin, and CCO. Moreover, Cu is essential for LOX activity, which is responsible for ECM remodeling during initiation and maturation of vascularization.

The maintenance of vascular homeostasis depends on the dynamic stability of the cells and ECM that constitute blood vessels. Cu is involved in the regulation of vascular cells and the ECM via many factors and enzymes, as depicted in Figure 5.

Oxidative stress disrupts vascular homeostasis by impairs ECs function and vascular wall. Cu is a constituent of superoxide dismutase (SOD) and ceruloplasmin (CP), both are importantly involved in preventing oxidative injury [175–178]. It was also shown that low dietary Cu intake reduces glutathione peroxidase activity [179, 180]. Cu is required for mitochondrial cytochrome c oxidase (CCO) activity, rendering it essential for oxidative phosphorylation [181]. Cu deficiency leads to depressed activity of CCO [124, 182, 183]. These abovementioned enzymes or proteins are so crucial for maintaining the homeostasis of vascular system [184–189].

Several *in vitro* studies have examined Cu redox activity [190, 191]. Cu is redox active and involved in ROS generation [191]. Exposure to elevated levels of Cu significantly decreases glutathione levels [192]. The depletion of glutathione may enhance the cytotoxic effect of ROS and allow the metal to be more catalytically active, thus producing higher levels of ROS [193]. However, it has been demonstrated that there is virtually no free Cu in the biological system [194]. Therefore, the redox injury generated from free Cu *in vitro* studies may not extrapolated to the *in vivo* conditions. A general belief is that Cu-related generation of ROS is more related to Cu overload [195–197], but it is Cu deficiency that causes severe oxidative stress partially resulting from mitochondrial respiration defects due to CCO depression [182].

The ECM is an important component of the blood vessel wall and is essential for maintaining the structural integrity of blood vessels. Cu is essential for the synthesis and maturation of ECM. Fibronectin, an avascular elongation promoter [198], was observed to be increased in cultured ECs in exposure to trace amounts of Cu [199]. Fibronectin mats were strengthened when a small amount of Cu was present [200]. Lysyl oxidase (LOX) is a critical enzyme involved in the ECM remodeling, and Cu is required for the LOX activity [201]. Elastin is required for the lumen formation and maintenance. Studies conducted in swine found that Cu is associated with aorta elastin and essential for the function of the vessels [202–204].

Therefore, Cu is essential for vascular homeostasis through its action on multiple enzymes or proteins involved in the regulation of vascular cells and ECM. In most cases, the disturbance of vascular homeostasis would result from Cu deficiency, although Cu overload also causes severe consequences in vascular homeostasis. In terms of atherosclerosis, Cu deficiency in the vascular tissue and Cu overload in circulation are often observed concomitantly, leading to a double damage to the vessel wall.

## Disturbance of Cu homeostasis in atherosclerosis

Cu concentrations were significantly reduced in atherosclerotic vascular walls [142]. High dietary cholesterol feeding causes hypercholesterolemia and atherosclerosis in animal models [139, 205]. During the process of fatty substances deposition and vascular wall hardening in large and medium-sized arteries [206], Cu concentrations in the atherosclerotic wall became significantly reduced compared to that in the normal aortic wall [139, 142]. This reduction in Cu concentrations leads to decreased proliferation and migration of endothelial cells, and inhibits the synthesis of ECM. Therefore, Cu deficiency has been postulated to be a triggering event of atherosclerosis in high cholesterol-fed animals, alongside multiple other hypotheses on the etiology of atherosclerosis induced by high dietary cholesterol [207–209].

Many studies from animal models to human clinical data reported that plasma Cu levels are significantly elevated along with hypercholesterolemia in atherosclerotic subjects [140, 141]. Two cross-sectional clinical studies with apparently healthy subjects showed that serum Cu was inversely associated with low-density lipoprotein cholesterol (LDL-C), suggesting that a higher or adequate serum Cu level is linked to a better lipid metabolic state [210]. It was interesting to note that the reduction of Cu in the atherosclerotic lesion vessels is associated with an increase in the serum Cu concentrations [138]. Although the reason for Cu loss in the atherosclerotic wall and increase in the serum are unknown, a recent study clearly demonstrated an inverse correlation between Cu concentrations in vascular plaque and the severity of atherosclerotic lesions [138].

In brief, Cu plays a crucial role in the process of vascular development and homeostasis. Maintaining Cu homeostasis is essential for maintaining vascular stability, including its regulation of ECs function and lipid metabolism, as well as combating oxidative stress. Numerous studies have shown that Cu homeostasis in vascular tissue is disturbed during the process of atherosclerosis, with Cu being lost from atherosclerotic plaques but increased in the plasma. This process is directly related to the severity of vascular disease. Therefore, we hypothesize that reversing treatment of atherosclerosis can be achieved by restoring Cu homeostasis in the diseased blood vessels.

## Reversal of atherosclerosis by restoring vascular Cu homeostasis

There have been numerous exploratory studies on the use of Cu supplementation for the treatment of atherosclerosis, although the results generated are uncertain and sometimes controversial. David et al [211] found that dietary Cu supplementation reduces atherosclerosis in the cholesterol-fed rabbit. Eman et al. found that Cu supplementation reduced cholesterol diet-induced atherosclerosis in rabbit [209]. However, a meta-analysis of 176 randomized controlled clinical trials showed no effect of Cu supplementation on lipid levels [212]. Similarly, another study found that dietary Cu supplementation had no significant effect on atherosclerosis or serum lipid levels in rats [213].

**FIGURE 6:**
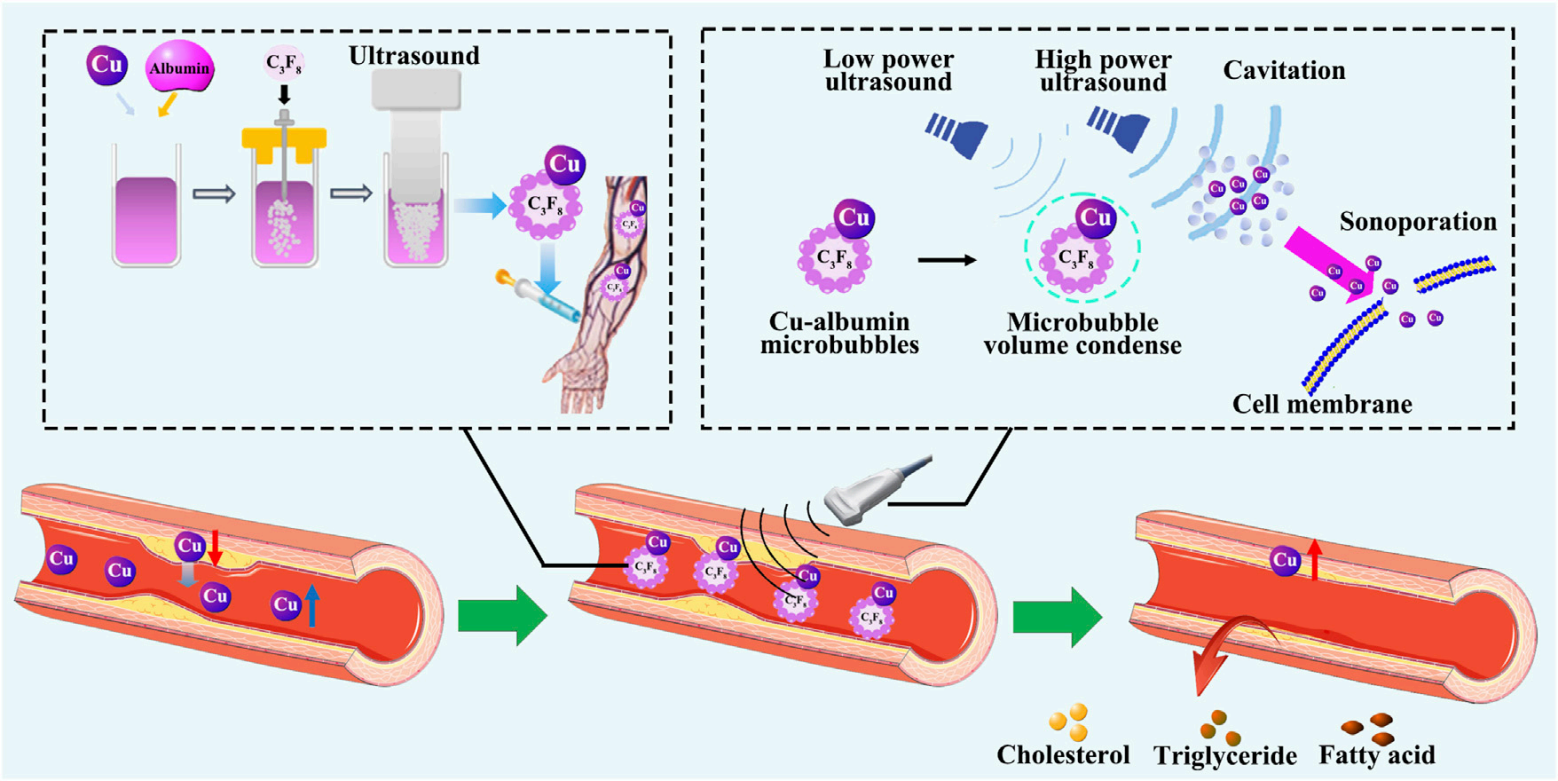
Reversal of atherosclerosis by restoring Cu homeostasis. Cu and albumin are prepared to make Cu-albumin microbubbles through ultrasonic vibration, and then injected by intravenous infusion. Under high-energy ultrasound, the microbubbles are broken, and Cu enters the diseased vascular tissue. After treatment with Cu-albumin microbubbles, the area of atherosclerotic plaques significantly decreases, Cu content in the diseased tissue significantly increases, the lipid content significantly decreases, the apoptosis of endothelial cells is reduced, and the stability of the plaque is not affected.

We made an effort to clarify possible differences among these confounding results. As shown in Figure 6, we fed laboratory rabbits a high-fiber diet supplemented with 1% (w/w) cholesterol for 12 weeks to create an atherosclerosis model [214]. Rabbits fed a cholesterol-supplemented diet had higher serum cholesterol levels and developed atherosclerosis. Cu concentrations in the cholesterol-fed rabbits were increased in the serum and kidney but decreased in the atherosclerotic lesion walls and multiple organs, including heart, liver, spleen, and lungs [138]. These results indicate that the body as a whole is not deficient in Cu during the pathogenesis of atherosclerosis caused by high cholesterol, but Cu homeostasis is altered, leading to unbalanced distribution of Cu to different organ systems [138]. Therefore, if a simple dietary Cu supplementation would only increase Cu levels in the blood, but would not help to replenish Cu content in the Cu deficient organ systems, including the atherosclerotic vessel walls.

There are studies showing that the increase in serum Cu levels can exacerbate LDL oxidation, which may lead to worsened atherosclerosis [215]. Thus, a simple dietary Cu supplementation approach would not be a reasonably feasible approach to replenish Cu in the Cu-deficient vessel wall due to the risk of adverse effects of further serum Cu elevation. The fundamental problem to be solved in the reversal of atherosclerosis by Cu supplementation is how to supplement Cu to the Cu deficient target organ systems.

To solve this problem, we developed an ultrasound-assisted Cu-albumin microbubbles (Cu-MB-US) target-specific Cu delivery procedure [215]. In this procedure, Cu was first reacted with albumin to form a Cu-albumin complex, followed by a microbubble formation through ultrasonic vibration. The Cu-albumin microbubbles were then injected intravenously into rabbits with atherosclerotic vessel lesions. High-energy ultrasound was used to irradiate the atherosclerotic lesion area. As the Cu-albumin microbubbles flowed through the area with blood, the high-energy ultrasound induced the collapse of the microbubbles, causing instant cell cavitation-directed penetration of Cu into the lesion tissue. This process achieved atherosclerotic lesion-specific Cu delivery.

The treatment with Cu-MB-US resulted in an average of 24.2% reduction in lesion surface area (from an average of 79.0% without treatment to an average of 54.8% after treatment). The use of pure albumin microbubbles demonstrated no therapeutic effect [215]. Furthermore, the treatment with Cu-MB-US did not increase Cu levels in plasma, but did significantly increase Cu levels in the diseased vascular tissue [215]. There was an inverse correlation between Cu concentration and the size of the atherosclerotic lesion [215]. Histopathological examination demonstrated that the reduction in atherosclerotic plaques was associated with a decrease in lipid content within the arterial wall after the Cu-MB-US treatment. Moreover, Cu repletion significantly reduced the cholesterol and phospholipid contents in the lesion tissue [215]. There was a positive correlation between cholesterol and phospholipid levels and the size of the atherosclerotic lesion. Consequently, Cu levels were inversely correlated with the levels of cholesterol or phospholipid in the lesion. Importantly, treatment with Cu-MB-US did not decrease the stability of atherosclerotic plaques. Cu-MB-US significantly reduced apoptosis of endothelial cells in the atherosclerotic lesion area. LOX activity and VSMC contents in the lesion were not altered after the treatment. Cu repletion did not alter the collagen content or the ratio of collagen I to collagen III in the lesion [138, 215].

In brief, it appears that Cu efflux from the atherosclerotic lesion walls during the pathogenesis of atherosclerosis leads to Cu deficiency in the injured vessel tissue and Cu elevation in the circulation. These double injuries generated from Cu deficiency in the affected organs and Cu overload in the circulation would not be recovered by a simple dietary Cu supplementation. Therefore, some confounding results from Cu supplementation on atherosclerosis, particularly between animal studies and human studies, would be explained at least partially by differential disease stages at which Cu supplementation was administrated. In the acute phase, Cu supplementation may show some beneficial effects, but in the late phase of chronic development, it may not be beneficial, or it may be adverse. It is thus reasonable to observe the beneficial effect of target-specific Cu delivery to the Cu-deficient lesion vessels, as reported recently [138, 215–217].

## Challenges for the reversal of atherosclerosis

Reversal of atherosclerosis is a new challenge in clinical practice. Studies in the last two decades have shifted the focus of treatment for atherosclerosis from on the cholesterol and thrombotic material deposition in the arterial wall to on the multifactorial inflammatory interactions. Based on the advanced understanding of the pathogenesis of atherosclerosis, it should be recognized that rejuvenating the self-repair mechanism of the vascular tissue is an appealing approach for the treatment of atherosclerosis [218]. Recent studies have shown that in the early stages of atherosclerotic lesions, the body has the ability to self-repair by removing the deposited lipids and repairing the injured vasculature; this has been demonstrated in many early clinical studies. However, as the pathological microenvironment of the diseased vascular tissue continues to deteriorate, the body gradually loses its self-repair ability, and lipid metabolism pathways become imbalanced, leading to further aggravation of the lesion and falling into a vicious cycle. Along with this process, Cu homeostasis is disturbed, further worsening the severity of atherosclerosis.

Restoring Cu homeostasis in the vessel wall may present a feasible treatment strategy for reversing atherosclerosis. By delivering Cu to the Cu-deficient lesion vascular tissue, it effectively reduced the area of atherosclerotic plaques [215]. While this procedure has proved to be successful in animal studies, its extrapolation to humans will face safety issues and adaptation modifications.

The treatment time window of atherosclerosis is crucial. Some patients can restore vascular health with exercise alone in the early stages of the disease. Studies showed that once vascular tissue lesions progress to foam cell formation and lipid stripes appearance, the disease often progresses irreversibly [219]. Inflammation-induced foam cell accumulation, cell apoptosis, necrosis, and subsequent formation of necrotic cores further aggravate the inflammatory response. Existing drug and surgical treatments are often applied until the disease progresses to a specific late stage, which cannot fundamentally change the progression of the disease. However, implantation of early treatment would require the development of new technologies instead of application of the existing technologies in the early stage of the disease, demanding innovations.

Vascular tissue lesions are a manifestation of the imbalance between organ injury and regeneration [220]. Treating only the factors that cause damage to the vessels has been proven to have limitations in the expected outcome. The development of future technologies should focus more on the restoration of regenerative capacity, including reversing the inflammatory phenotype of ECs and promoting the efflux of lipids from the vascular wall, which has already been carried out in many preclinical studies. From the perspective of promoting the restoration of the injury-repair mechanism and rebuilding the body’s autonomous regenerative capacity, the development of earlier intervention treatment for atherosclerosis would achieve better reversal treatment results. This not only requires continuous breakthroughs in new technologies but also requires a shift in the medicinal concept from disease treatment to health rejuvenation. The reversal of atherosclerosis is possible, but we have to face new challenges for its fulfilment in the future.
